# Exposure to ultraviolet radiation during pregnancy influences offspring immune and inflammatory responses in Wistar-strain rats

**DOI:** 10.5935/1518-0557.20220059

**Published:** 2023

**Authors:** Kolawole O. Adio, Bernard O. Adele, Abayomi O. Ige, Elsie O. Adewoye

**Affiliations:** 1 Applied and Environmental Physiology unit, Department of Physiology, University of Ibadan, Nigeria

**Keywords:** ultraviolet radiation, pregnancy, immune response, inflammation

## Abstract

**Objective:**

During pregnancy, maternal exposure to ultraviolet radiation (UVR) has been linked to altered offspring immune and health status. This study was therefore designed to investigate some markers of immune response in the offspring of pregnant Wistar rats exposed to UVR at various points of gestation.

**Methods:**

Thirty pregnant rats were divided into 6 groups (n=5) as follows; group I, control, consisting of pregnant rats unexposed to UVR. Animals in groups II, III, IV, V and VI were exposed to UVR for one hour daily, on gestational days 1-7,8-14,15-21,1-14 and 1-21, respectively. Animals were allowed to come to term and offspring birth weight was taken. On postnatal Day 10, weight of each offspring was taken again. Thereafter, blood samples were collected from each offspring per group and evaluated for total protein, albumin, globulin, C-reactive protein, interleukin-1β, and complement component protein-3 (C3). Offspring hepatic samples were evaluated using standard histological techniques.

**Results:**

Offspring birthweight increased (*p*<0.05), while weight gain on postnatal day 10 reduced in all experimental groups compared to controls. No significant differences were observed for offspring total protein, albumin, and C3 levels across all groups. Globulin increased (*p*<0.05) only in group VI, while C-reactive protein increased (*p*<0.05) in all experimental groups, except group III, compared to controls. Interleukin-1β in groups II, III, V and VI increased significantly compared to controls. Offspring hepatic samples exhibited hepatocellular degeneration and necrosis that was independent of gestational stage of maternal exposure to UVR.

**Conclusions:**

Maternal exposure to ultraviolet radiation during gestation in Wistar rats activates offspring immune and inflammatory responses.

## INTRODUCTION

The depletion of the ozone layer on a global scale has been observed and this has been attributed to increases in halocarbon emissions (Slaper *et al.*, 1996; Angell & Korshover, 2005), unregulated rocket launches (Sivasakthivel & Reddy, 2011), global warming (Last, 1993), and increased emission of nitrogenous compounds (Ravishankara *et al.*, 2009). This depletion of the ozone layer decreases the earth’s shield against harmful ultraviolet (UV) rays that radiate from the sun, hence increasing human exposure. Ultraviolet (UV) radiation can be defined as a form of electromagnetic radiation that comes from the sun and man-made sources like tanning beds and welding torches (Boniol *et al.*, 2012). There are three main types of UV radiation, namely UVA (400- 320 nm), UVB (320-290 nm), and UVC (290-200 nm). The strength at which these ultraviolet radiations reach the surface of the earth has been reported to depend on time of the day, altitude, and season (NTP, 2021). Excessive exposure to UV rays has been reported to result in various deleterious conditions such as skin cancers, cataracts and other eye diseases, as well as accelerated skin ageing (Wehner *et al.*, 2012). However, studies have also shown that moderate exposure to ultraviolet radiation causes an increase in vitamin D activity, which is important in the regulation of calcium metabolism, blood pressure, and mediating immunity as well as immune responses in the body (Wacker & Holick, 2013).

In pregnancy, studies have shown that proper growth, development and likely presence of disease in the fetus is correlated to weight, pregnancy duration and geographical location (Barker, 1995; 2000; Godfrey & Barker, 2000). Studies have also established that a relationship between premature birth and low birth weight exists with the environmental conditions during fetal life, maternal and fetal immune systems, infections, and vitamin D status (Karras *et al.*, 2016; Megaw *et al.*, 2017). Furthermore, increased maternal exposure to UV rays during pregnancy has also been associated with an increase in the development of multiple sclerosis and schizophrenia in adults (Botyar & Khoramroudi, 2018). These observations suggest that there may be a link between maternal exposure to UV rays, neonatal homeostasis and immune responses.

Acute-phase proteins are part of the innate immune response system and their general function is related to defense against pathological damage and restoration of homeostasis (Jain *et al.*, 2011). They are plasma proteins synthesized in the liver and their concentrations have been reported to often increase (or decrease) by 25% or more during inflammation and infections (Jain *et al.*, 2011). These proteins serve as inhibitors or mediators of the inflammatory process and include albumin, C-reactive protein, α1-acid glycoprotein, haptoglobin, mannose-binding protein, fibrinogen, α1-antitrypsin, and complement components C3 and C4 (Ackermann, 2017). These proteins are an integral part of the acute phase response that results in a systemic complex reaction with the objective of reestablishing homeostasis and promote health (Cray *et al.*, 2009).

Though some studies have indicated that exposure to UV rays during pregnancy may exert some benefits, it is unclear with the continued depletion of the ozone layer and hence increased exposure to UV rays, whether these benefits would outweigh the reported harmful effects of UV ray exposure. This study was therefore designed to investigate some inflammatory markers and acute phase response proteins in the offspring of pregnant Wistar rats that were exposed to ultraviolet radiation.

## MATERIALS AND METHODS

### Animal, grouping and experimental protocol

Thirty female Wistar rats weighing (100-120g) were housed in well aerated cages, fed on standard animal chow, given free access to drinking water, and exposed to natural atmospheric condition, room temperature and alternating day and night cycles. They were acclimatized in the animal house prior to commencement of experimental procedures. The animals were maintained under humane conditions in accordance with guidelines laid down by the Animal Care and Use Research Ethics Committee, University of Ibadan and that of the Guide for the Care and Use of Laboratory Animals (NRC, 1996), published by National Academy Press, 2101 Constitution Ave. NW, Washington, DC 20055, USA. After fourteen days of acclimatization, female animals in the proestrus phase of the estrous cycle were identified by taking and monitoring vaginal smears daily. These female animals were allowed to mate with male animals and the presence of sperm in the vaginal smear was taken as an indicator of pregnancy and day 1 of the gestational period in the female rats. Thereafter, animals were grouped into 6 groups of 5 animals each as follows; group I was control and consisted of pregnant dams not exposed to UV rays. Pregnant animals in groups II, III, IV, V and VI where exposed daily to ultraviolet radiations for one hour (9am-10am) on gestational days 1-7, 8-14, 15-21, 1-14 and 1-21, respectively.

### Exposure to ultraviolet radiation protocol

Animals in the exposure groups were transferred every morning to a wooden exposure chamber (90 x 60 x 90 cm) that had Ultraviolet (UV) emitting bulbs - UV A (Sylvania blacklight F15W/350BL-T8 (5W, 350nm), UV B (Sankyo Denki G15T8E (15W, 312nm) and UV C (Ultraviolet G15 Mass (15W, 254nm) - attached to its roof, each connected to a power source. The bulbs were switched on 15 minutes after the animals were transferred to each chamber. The animals were simultaneously exposed to UV A, B and C for one hour, respectively. Thereafter, the animals were transferred back to their cages and maintained for the rest of the day under normal animal house conditions as previously stated. Animals in the control group were also daily transferred to similar exposure chambers for the same duration however, the UV emitting bulbs were not switched on. Thereafter, the control animals were also transferred back to their cages and exposed to normal animal house conditions for the rest of the day.

### Blood collection and biochemical analysis

The animals were allowed to come to term and the birth weights of their offspring were taken. On postnatal day 10, the weight of each pup was taken per group and the pups were placed in a glass fume chamber containing cotton wool soaked with diethyl ether, as anaesthetic agent, for 5 minutes. Blood samples were obtained from each pup per group by cardiac puncture into EDTA-laced sample bottles, allowed to stand at room temperature and thereafter centrifuged at 3500rpm for 10 mins to separate out plasma, which was evaluated for C-reactive protein, interleukin 1β, complement component 3 (C3), total protein, albumin and globulin levels, respectively, using commercially available ELISA kits. The pups were subsequently euthanized by returning them to the diethyl ether-filled fume chamber for another 15 minutes. Thereafter, the whole liver was excised from each pup, weighed and structural histological changes therein were evaluated using Haematoxylin and Eosin (H and E) stains.

### Statistical analysis

Data obtained are expressed as mean ± SEM and statistical difference within and between the groups were evaluated using ANOVA and the Mann-Whitney U post-hoc test. Statistically significant difference between groups was taken at *p*<0.05.

## RESULTS

### Weight changes in the offspring of control and experimental groups

There was a significant increase (*p*<0.05) in the mean birthweight in groups II (45.9%), III (57.9%), IV (43.8%), V (36.1%) and VI (48.4%) compared to controls (group I). No significant difference in body weight was observed on day 10 in the experimental groups when compared with controls. However, percent weight gains on day 10 within each experimental group (II-VI) were 45.9%, 53.1%, 46.7%, 45.8%, and 37.6%, all lower than the gain observed in the control group (Table 1).

**Table 1:** Effect of maternal ultraviolet ray exposure on offspring weight changes in control and experimental groups.

Groups	Mean birth Weight (g)	Body weight on day 10 (g)	Percent weight gain on day 10 within each group (%)
I	3.68±0.01	13.72±0.34	272.8
II	5.37±0.17^*^	13.36±0.45	148.8^*^
III	5.81±0.19^*^	13.33±0.32	129.4^*^
IV	5.29±0.02^*^	13.04±0.50	146.5^*^
V	5.01±0.38^*^	12.58±0.93	151.1^*^
VI	5.46±0.10^*^	14.80±0.18β	171.1^*^

Values are mean ± SEM

* indicates values that are significantly different from controls (group I) at *p*<0.05. I = Control group; II = Maternal UV exposure group on gestational day 1-7; III = Maternal UV exposure group on gestational day 8-14; IV = Maternal UV exposure group on gestational day 15-21; V = Maternal UV exposure group on gestational day 1-14, VI = Maternal UV exposure group on gestational day 1-21.

### Liver weight and plasma protein level in control and experimental groups

There was no significant difference in liver weight, total protein, and albumin levels in the experimental groups when compared with controls. However, globulin levels (mg/dL) in groups IV (3.23±0.20), V (2.65±0.05), and VI (3.42±0.44) were significantly increased (*p*<0.05) compared to group I (1.99±0.18) (Table 2).

**Table 2:** Effect of maternal ultraviolet ray exposure on offspring liver weight and plasma protein levels in control and experimental groups.

Groups	Liver weight (g)	Total protein (mg/dL)	Albumin (mg/dL)	Globulin (mg/dL)
I	0.14±0.01	5.32±0.29	3.51±0.40	1.81±0.11
II	0.13±0.00	5.25±0.17	3.26±0.29	1.99±0.18
III	0.15±0.01	5.19±0.22	3.23±0.70	1.96±0.58
IV	0.13±0.01	6.03±0.20	2.81±0.46	3.23±0.20^*^
V	0.13±0.02	5.72±0.26	3.07±0.21	2.65±0.05^*^
VI	0.15±0.01	6.04±0.49	2.66±0.05^*^	3.42±0.44^*^

Values are mean ± SEM

* indicates values that are significantly different from controls (group I) at *p*<0.05I = Control group; II = Maternal UV exposure group on gestational day 1-7; III = Maternal UV exposure group on gestational day 8-14; IV = Maternal UV exposure group on gestational day 15-21; V = Maternal UV exposure group on gestational day 1-14, VI = Maternal UV exposure group on gestational day 1-21.

### C-reactive protein, Interleukin 1β and Complement component 3 levels in control and experimental groups

**Figure 1:**
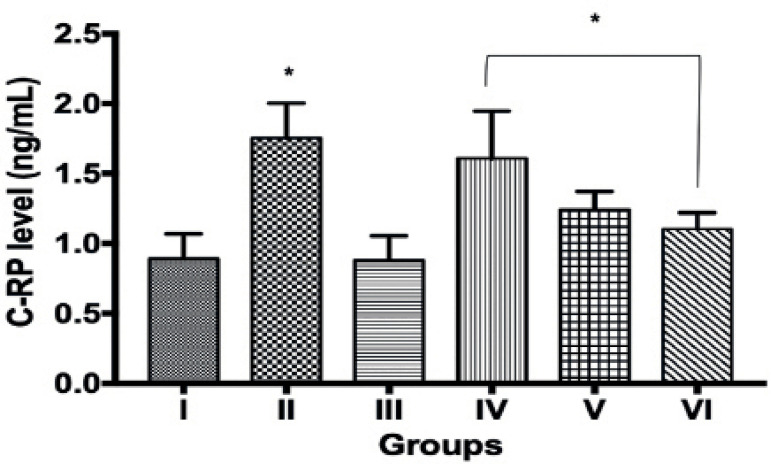
C-reactive protein level in control and experimental groups. Values are mean ± SEM. * indicates values that are significantly different from controls (group I) at *p*<0.05. I = Control group; II = Maternal UV exposure group on gestational day 1-7; III = Maternal UV exposure group on gestational day 8-14; IV = Maternal UV exposure group on gestational day 15-21; V = Maternal UV exposure group on gestational day 1-14, VI = Maternal UV exposure group on gestational day 1-21.

**Figure 2:**
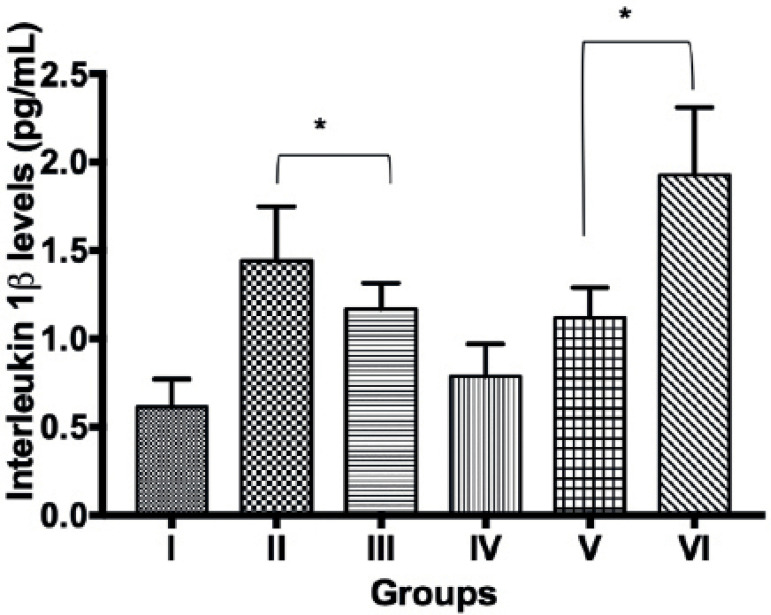
Interleukin 1 β level in control and experimental groups. Values are mean ± SEM. * indicates values that are significantly different from controls (group I) at *p*<0.05. I = Control group; II = Maternal UV exposure group on gestational day 1-7; III = Maternal UV exposure group on gestational day 8-14; IV = Maternal UV exposure group on gestational day 15-21; V = Maternal UV exposure group on gestational day 1-14, VI = Maternal UV exposure group on gestational day 1-21.

**Figure 3:**
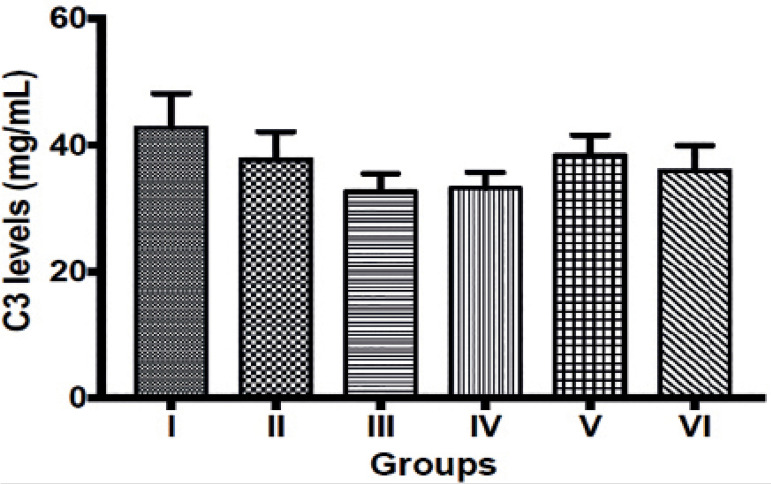
Complement component 3 levels in control and experimental groups. Values are mean ± SEM. I = Control group; II = Maternal UV exposure group on gestational day 1-7; III = Maternal UV exposure group on gestational day 8-14; IV = Maternal UV exposure group on gestational day 15-21; V = Maternal UV exposure group on gestational day 1-14, VI = Maternal UV exposure group on gestational day 1-21.

There were significant increases (*p*<0.05) in C-reactive protein (CRP) levels (ng/mL) in groups II (1.75±0.09), IV (1.61±0.13), V (1.24±0.05) and VI (1.10±0.04) when compared to group I (0.89±0.07). No significant difference was observed in CRP values between groups III and I (Figure 1). Interleukin 1β in group II (136.1%), III (91.8%), V (83.6%) and VI (216.4%) were significantly increased (*p*<0.05) compared to group 1. However, interleukin 1β levels (pg/mL) observed in group IV (0.76±0.07) was comparable with that in group I (0.61±0.06) (Figure 2). No significant difference was observed in complement component 3 levels between the control group (group I) and other experimental groups (Figure 3).

### Histological evaluation of offspring liver samples in control and experimental groups

**Figure 4:**
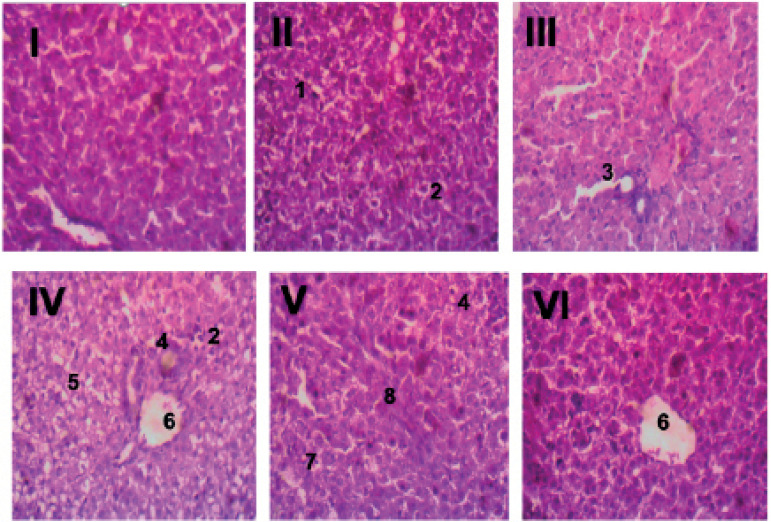
Histology of the liver. Liver samples of control animals (I) showed well preserved hepatic structural architecture and normal hepatocytes with no observable lesions. Animals in group II (day 1-7 UV maternal exposure group) exhibited liver samples with multifocal hepatocellular degeneration (1) and coagulation necrosis (2). Liver samples from group III animals (day 8-14 UV maternal exposure group) showed moderate centrilobular hepatocellular degeneration. Group IV (day 15-21 UV maternal exposure group) had liver tissues with periportal vacuolar hepatocellular degeneration (5), coagulation necrosis (2), a few foci of inflammation (4) and centrilobular hepatocellular necrosis (6). In group V (day 1-14 UV maternal exposure group), liver samples showed random hepatocellular coagulation necrosis (7), inflammation (4) and fibroblast proliferation (8). Animals in Group VI (day 1-21 UV maternal exposure group) also exhibited centrilobular hepatocellular coagulation necrosis (6).

An assessment of offspring liver samples is shown in Figure 4 (I-VI). Liver samples of control animals (group I) showed well preserved hepatic structural architecture and normal hepatocytes with no observable lesions. Animals in group II (day 1-7 UV exposure group) exhibited liver samples with multifocal hepatocellular degeneration and coagulation necrosis. Liver samples from group III animals (day 8-14 UV exposure group) showed moderate centrilobular hepatocellular degeneration. Group IV (day 15-21 UV exposure group) had liver tissues with periportal vacuolar hepatocellular degeneration, coagulation necrosis, a few foci of inflammation and centrilobular hepatocellular necrosis. In group V (day 1-14 UV exposure group), liver samples showed random hepatocellular coagulation necrosis, inflammation and fibroblast proliferation. Animals in Group VI (day 1-14 UV exposure group) also exhibited centrilobular hepatocellular coagulation necrosis.

## DISCUSSION AND CONCLUSION

Natural ultraviolet (UV) rays originate from the sun. Artificial sources are also produced for use in industry, commerce and recreation. As previously highlighted, UV rays are classified into three types, namely UVA, UVB and UVC (Boniol *et al.*, 2012). When light from the sun passes through the atmosphere, approximately 90% of UVB and all of UVC radiation is absorbed by the ozone layer, water vapor, oxygen and carbon dioxide (Tatalovich *et al.*, 2006). Hence, the larger portion of UV rays that reach the surface of the earth is made up mostly of UVA and (to a smaller extent) UVB (Tatalovich *et al.*, 2006). However, with the increasing depletion in the thickness of the ozone layer by human created pollution activities, the amount of UVA, UVB and UVC reaching the surface of the earth has increased, and this has impacted the health of humans, animals, marine organisms and plant life (Anwar *et al.*, 2016). In humans, increased exposure to UV rays has been associated with skin cancer, cataracts and immune system damage (NTP, 2021). The immune system is the host defense mechanism that prevents and protects the body from invasion by foreign pathogens. It is made up of two systems, the innate and the adaptive immune system, which closely interact with each other (Marshall *et al.*, 2018).

Several studies in humans and animals have associated birth weight with the health status of offspring (WHO, 2014). Low birth weight has also been positively correlated with maternal nutritional status and insults (Calkins & Devaskar, 2011; Englund-Ögge *et al.*, 2019), which often results in offspring immature immune system and small sized lymphoid organs (Raqib *et al.*, 2007). This study showed an increase in birthweight of all offspring from maternal rats exposed to UV rays, regardless of whether the UV exposure was early (1-7day), midway (8-14days), or late (15-21days) in the gestational period (Table 1). This is consistent with some studies (Palacios *et al.*, 2016) that have ascribed this observation to increased activation of vitamin D by UV rays resulting in the formation of 25, hydroxy cholecalciferol (25OH-VitD), which in turn is converted to 1, 25, dihydroxycholecalciferol (1.25OH-VitD) (in the kidney), resulting in an increase in calcium absorbance, bone growth, density and strength as well as an increase in birthweight (Wei *et al.*, 2013). However, on post-natal day 10, weight in the control and experimental groups was comparable, suggesting a decline in growth rate in the experimental group compared to controls (Table 1). This, again, is in accordance with the reports of Geldenhuys *et al.* (2014) and Fleury *et al.* (2016) who, while carrying out anti-obesity studies, reported a decline in weight gain in animals exposed to UV radiation and suggested that this could be attributed to an increase in circulating vitamin D3 level as a result of increased exposure to UV rays, which has been associated with reduced weight gain in both animal (Fleury *et al.*, 2016) and human studies (LeBlanc *et al*., 2012).

The liver, though not traditionally classified as an immunologic organ, performs many essential immune tasks, some of which include the induction of immune tolerance and strong innate immunity (Racanelli & Rehermann, 2006). Hepatocytes are responsible for the production of 80-90% of the circulating innate immunity proteins in the body and also contain a large number of resident immune cells that are constantly exposed to a wide variety of bacterial products, environment toxins, and food antigens (Gao *et al.*, 2008). In toxicology studies, the physical and histological examination of the liver has been identified as being key to the understanding of the toxicological effects and implications of experimental or natural exposure to drugs and/or chemical agents (Cattley & Cullen, 2013). Liver weight changes have been suggested as a useful tool in detecting and quantitating the effects of hepatotoxins. Decreases in liver weight reflect loss in functional mass associated with atrophy or significant lethal hepatocellular injury, while increases in liver weight suggest generalized accumulations and adaptive changes such as hypertrophy and/or hyperplasia (Cattley & Cullen, 2013). There was no significant difference in liver weight between the offspring of maternal UV exposed groups to the offspring of controls, which suggests absence of immediately apparent hepatic toxicity in both groups. However, histological evaluation of offspring liver samples shows pathologies that appear consistent with mild hepatic damage and infection (Figure 4), which appeared to be independent of the gestational stage at which maternal exposure to UV rays occurred.

The determination of total plasma protein, a routine health test, measures the total protein, specifically albumin and globulin levels, in blood. There was no significant difference in total protein concentration between control and experimental groups. However, albumin, an acute phase protein and the major protein constituent of total serum proteins, was reduced in the offspring of pregnant dams exposed to UV rays throughout the gestational period (group VI), suggesting the likely presence of malnourishment or inflammation (Don & Kaysen, 2004) in this group. Furthermore, offspring from groups IV, V, and VI (maternal UV exposure on gestational days 15-21, 1-14 and 1-21, respectively) exhibited increased globulin levels suggesting the presence of infection, inflammation and activation of the immune system (O’Connell *et al.*, 2005).

Exposure to UV rays has been described as one of the most potent inducers of cytokine release (Schwarz & Luge, 1989) resulting in local and systemic immunologic and inflammatory reactions (Shreedhar *et al*., 1998). Increased maternal cytokine production has also been reported to affect neonatal inflammatory response (Schwarz & Luge, 1989; Hsiao & Patterson, 2011), resulting in an increase in neonatal cytokine production (Hsiao & Patterson, 2011). Cytokines, especially interleukin-1beta (IL-1β) and interleukin-6 (IL-6), are involved in the acute phase response and have been reported to stimulate the secretion of C-reactive protein (CRP) from the liver (Bermudez *et al.*, 2002; Kramer *et al.*, 2008). This study showed increases in IL-1β in the offspring of all UV maternal exposure groups except group IV, where values though increased, were not significantly different from controls (Figure 2). This increase in IL-1β could be ascribed to UV exposure-induced maternal cytokine production, which might have been transferred to the fetus resulting in the stimulation of fetal immune system, immunosuppression, and activation of inflammatory processes. Furthermore, increased CRP levels were also observed in the offspring from all groups except group III (UV maternal exposure group on gestational days 8-14) (Figure 1), suggesting the likely presence of trauma, inflammation, and infection in these groups. However, complement component protein 3 (C3), an important innate immune response protein that helps to kill bacteria and viruses that cause diseases (Dunkelberger & Song, 2010), was not significantly different across the groups, suggesting that at the time of sample collection, there was no bacterial or viral infection in these groups despite an increase in markers of inflammation and immuno-suppression.

As previously mentioned, UV rays from the sun are mainly composed of three types UV rays A, B and C (Boniol *et al.*, 2012). Of these three, UVA and part of UVB is what gets to the surface of the earth. These rays, in mild to moderate proportions, have been reported to exert beneficial effects on human, animal, and plant health (Wacker & Holick, 2013). Studies have suggested that most of the deleterious effects ascribed to UV exposure may actually be due to increased exposure to UVB and UVC (D’Orazio *et al.*, 2013). With the increase in the depletion of the ozone layer following human-pollution related activities, living things are increasingly getting exposed to UVA, UVB, and UVC (Bais *et al.*, 2018). This study attempted to mimic ozone depletion and increased exposures to UV rays, especially UVB and UVC, simultaneously during pregnancy, and has demonstrated alterations in the innate immune system of offspring following maternal exposure to these rays at various stages of pregnancy. It is likely that these observations may be associated with maternal activation of proinflammatory mediators as a result of increased exposure to UV rays, especially UVB and UVC, which subsequently affected fetal immune and inflammatory responses. However, the effects of maternal exposure to each type of UV ray on maternal and offspring immune responses was not investigated in this study. Although this is a limitation in the present study, it will form the crux of subsequent investigations in our laboratory.

In conclusion, this study suggests that maternal exposure to ultraviolet radiations (UVA, UVB and UVC) during gestation may activate maternal immune and inflammatory responses that can be transferred to offspring resulting in the modulation of the innate immune system and proinflammatory cytokine release in the offspring.
